# Fine structure of the compound eyes of the crepuscular moth *Grapholita molesta* (Busck 1916) (Lepidoptera: Tortricidae)

**DOI:** 10.3389/fphys.2024.1343702

**Published:** 2024-02-02

**Authors:** Xiaofan Yang, Hongfan Ran, Yueli Jiang, Ziyun Lu, Guoshu Wei, Jiancheng Li

**Affiliations:** ^1^ Institute of Plant Protection, Hebei Academy of Agricultural and Forestry Sciences, Key Laboratory of IPM on Crops in Northern Region of North China, Ministry of Agriculture and Rural Affairs of China, Baoding, Hebei, China; ^2^ Institute of Plant Protection, Henan Academy of Agricultural Sciences, Zhengzhou, Henan, China; ^3^ College of Plant Protection, Hebei Agricultural University, Baoding, Hebei, China

**Keywords:** Tortricidae, compound eye, ultrastructure, distal rhabdom, TEM

## Abstract

Morphological organization, ultrastructure and adaptational changes under different light intensities (10000, 100, 1, and 0.01 mW/m^2^) of the compound eye of the oriental fruit moth *Grapholita molesta* (Busck 1916) were investigated. Its superposition type of eyes consist of approximately 1072 ommatidia in males and 1029 ommatidia in females with ommatidial diameters of around 15 μm. Each ommatidium features a laminated corneal lens densely covered by corneal nipples of 256 nm in height. Crystalline cones are formed by four cone cells, proximally tapering to form a narrow crystalline tract with a diameter of 1.5 μm. Eight retinula cells, two primary and six secondary pigment cells per ommatidium are present. The 62.3 μm long rhabdom is divided into a thin 1.8 μm wide distal and a 5.2 μm wide proximal region. Distally the fused rhabdom consists of the rhabdomeres of seven retinula cells (R1-R7) and connects with the crystalline cone. In the proximal rhabdom region, the pigment-containing retinula cell R8 occupies a position in centre of the rhabdom while R1-R7 cells have taken peripheral positions. At this level each ommatidial group of retinula cells is surrounded by a tracheal tapetum. In response to changes from bright-light to dim-light adaptations, the pigment granules in the secondary pigment cells and retinula cells migrate distally, with a decrease in the length of crystalline tract.

## 1 Introduction

Compound eyes are the main visual receptor organs of insects. They consist of optical units known as ommatidia, which anatomically and optically conform to either the apposition and superposition type of eye (Exner, 1891). Ommatidia are made up of a cornea beneath which one finds the crystalline cone that is surrounded by primary and secondary pigment cells and in contact with the light-perceiving rhabdom in the centre of the cluster of retinula cells (Warrant and McIntyre, 1993). The retinula cells penetrate the basal matrix as axons. Despite such structural uniformity, reviewed by Meyer-Rochow (1999), different optical strategies under a variety of environmental conditions have led to the evolution of structural modifications to the eye, allowing to serve the insect in tasks of orientation, flight and migration, food procurement, obstacle and mate detection under both dim and bright photic conditions (Land, 1997; Cronin et al., 2014).

In Lepidoptera the apposition eye, in which there is no screening pigment free zone interposed between dioptric and perceiving structures of the eye, is a typical characteristic of most daytime-active butterflies (Yagi and Koyama, 1963; Warrant and Nilsson, 2006), while the considerably more light-sensitive insects such as crepuscular or nocturnal moths possess superposition eyes that can greatly increase photon capture and thus achieve a much higher optical sensitivity (up to 100–1000 times) than the apposition eyes of butterflies (Warrant and Dacke, 2011). Previous studies have shown that certain genera of micromoths, e.g., the nepticulids *Ectoedemia* (Honkanen and Meyer-Rochow, 2009) and *Stigmella* (Fischer et al., 2014), the argyresthid *Argyresthia* (Fischer et al., 2014), and the gracillarid *Cameraria* (Fischer et al., 2012; 2014) have evolved an intermediate eye type, a structural modification to the classical superposition eyes. A similar morphology that the distal rhabdom distally connected with the crystalline cone has also reported in some medium-sized moths such as the pyralid genera *Ephestia* (Horridge and Giddings, 1971) and *Amyelois* (Bernard et al., 1984), the noctuid *Spodoptera* (Meinecke, 1981), the crambid *Ostrinia* (Belušič et al., 2017; Chen et al., 2019) and the tortricid *Adoxophyes* (Hämmerle and Kolb, 1987; Satoh et al., 2017). Theses modified compound eyes are probable generalized as a consequence of adaptation to miniaturization or lifestyle.

The crepuscular oriental fruit moth *Grapholita molesta* (Busck) (Lepidoptera: Tortricidae), is a major pest of stone and pome fruits causing considerable economic damage worldwide (Rothschild and Vickers, 1991). Active mostly around dusk, when daylight intensity drops by five to six log units within a short period, this moth is widely assumed to use its olfactory system to discriminate between host and non-host plants (Natale et al., 2003; Piñero and Dorn, 2009; Lu et al., 2012; Najar-Rodriguez et al., 2013; Li et al., 2019; Chen et al., 2020). It has however been observed that adult *Grapholita molesta* are attracted to light, preferring violet and green light (Sun et al., 2014). Our recent works show that *G. molesta* can visually discriminate between plants in microhabitats to guide oviposition even in dim light, exhibiting a preference for higher brightness/intensity (Yang et al., 2020; 2022). Electroretinogram (ERG) spectral sensitivity measurements additionally reveal that three photoreceptor classes are present in compound eyes of *G. molesta*, with sensitivities peaking at UV (355 nm), blue (440 nm) and green (525 nm) wavelengths (Martín-Gabarrella et al., 2023). These visual behavior and physiology of *G. molesta* require a highly sensitive visual system, which is why we were interested in the structure of its compound eye as a first step in exploring the possibly adaptions to the changing light intensity at dusk.

In this paper we describe the morphological organization, ultrastructure and adaptational changes under different light intensities (10000, 100, 1, and 0.01 mW/m^2^, corresponding to daylight, early twilight, late twilight and moonlight, respectively) of the compound eye of adult *G. molesta* by scanning and transmission electron microscopy.

## 2 Material and methods

### 2.1 Insect collections and different light intensity adaptational treatments

Adult *G. molesta* were cultured from a laboratory strain derived from wild-caught larvae from a peach orchard in Shunping County (38°50′N, 115°8′E), Hebei Province, China in August 2020, and kept in an artificial climate chamber (GXZ-300B, Ningbo Southeast Instrument Co., LTD., China) at 26°C under light/dark cycles of 15L:9D (scotophase lasting from 19:00–4:00). Three-day-old males and females specimens were collected for further processing (see below).

For different light intensity adaptational experiments, specimens were exposed to four different light intensities (10000, 100, 1, and 0.01 mW/m^2^, corresponding to daylight, early twilight, late twilight and moonlight, respectively) for 2 h prior to decapitation at 20:00 (when the moths are mostly active). Specimens of four adaptational states were anesthetized and decapitated under their corresponding light intensities, respectively. A red LED lamp (*λ*_max_ = 630 nm) was used to provide enough illumination to decapitate the dimlight-adapted eyes (i.e., 0.01 mW/m^2^) of adult *G. molesta* without altering their adaptation state. Light intensity was measured at the half centre of the chambers using a radiometer (IL1700, International Light Research, Peabody, MA, USA) and adjusted by neutral density filters (Melles Griot, Rochester, NY, USA) and slightly lowering height of LED lamp.

### 2.2 Transmission electron microscopy (TEM)

Five specimens of four light intensity adaptational states (10000, 100, 1, and 0.01 mW/m^2^) of each sex were used for TEM. After decapitation, the heads were immediately fixed in a mixture 2.5% glutaraldehyde and 2.0% paraformaldehyde in phosphate buffered saline (PBS, 0.1 M, pH 7.4) at 4°C for 24 h. The fixed heads were rinsed in PBS and post-fixed in 1% osmium tetroxide (OsO_4_) in PBS at 4°C for 2 h. Following postfixation, the samples were rinsed in deionized distilled water (ddH_2_O, three shifts of 10 min at 4°C) and dehydrated using a graded ethanol series (30%, 50%, 70%, 80% and 90% for 10 min each and 100% for 30 min twice). Following infiltration with acetone/Epon mixtures (3:1, 1:1, 1:3, pure Epon), the samples were embedded in pure Epon 812 and polymerized at 45°C for 24 h and 60°C for 48 h.

For TEM observations, 70 nm thick serial longitudinal and transverse sections of ommatidia of the compound eye were cut with a diamond knife on a Leica EM UC6 ultramicrotome (Leica, Nussloch, Germany). The ultra-thin sections were stained with 2% uranyl acetate and 0.5% lead citrate and examined under a FEI Tecnai Spirit transmission electron microscope (FEI, Oregon, USA) operated at 120 kV.

#### 2.3 Scanning electron microscopy (SEM)

For SEM observations, the fixed heads (5 females and 5 males) were ultrasonically cleaned for several seconds after rinsing several times with PBS. Subsequently, samples were dehydrated using a graded ethanol series (30%, 50%, 70%, 80% and 90% for 10 min each and 100% for 30 min twice) and critical point dried in liquid carbon dioxide (Leica EM CPD300, Leica Microsystems, Wetzlar, Germany). Dried samples were mounted on SEM stubs using a double graphite adhesive tape and sputter-coated with gold at 10 mA current for 30 s to an approximate thickness of 20 nm (KYKY SBC-12, KYKY Technology Co., LTD., Beijing, China), and examined in a Hitachi SU8010 scanning electron microscope (Hitachi, Tokyo, Japan), operated at 5 kV.

### 2.4 Facet number and size

To study numbers and sizes of the facets, the eyes of adult *G. molesta* (5 males and 5 females) were removed from the heads and covered with a thin layer of colourless nail polish to produce cornea replicas (Ribi et al., 1989). Once dry, the replicas were carefully removed and flattened on a microscope slide by making incisions with a micro-scalpel. The replicas were coated with coverslips, sealed with melted wax, and photographed under an Olympus BX63 light microscope (Olympus, Tokyo, Japan).

### 2.5 Morphometric analyses

SEM micrographs were used to determine the dorso-ventral and antero-posterior lengths of the eye, the diameters of the facet (i.e., the corner to corner distance of the hexagonal corneal surface) and ocelli. The exact number of ommatidia per eye was measured on cornea replicas. Longitudinal sections for TEM were used to measure the ommatidial lengths, rhabdom lengths, radii of curvature of eye, corneal thicknesses, corneal facets, cone lengths and interommatidial angles (Schwarz et al., 2011; Fischer et al., 2012). Distal and proximal rhabdom diameters, the diameter of the microvilli, the diameters of pigment granules of primary pigment cells (PPC) and secondary pigment cells (SPC) as well as retinula cells were gathered from TEM transverse sections (Fischer et al., 2012). All measurements were analyzed using the ImageJ software (Rasband, W.S, U.S. National Institutes of Health, Bethesda, MD). The independent samples *t*-test was used to examine the difference between both sexes. All statistical analyses were performed using IBM-SPSS v.19.0 (IBM, Armonk, NY, USA).

## 3 Results

### 3.1 External morphology

**FIGURE 1:**
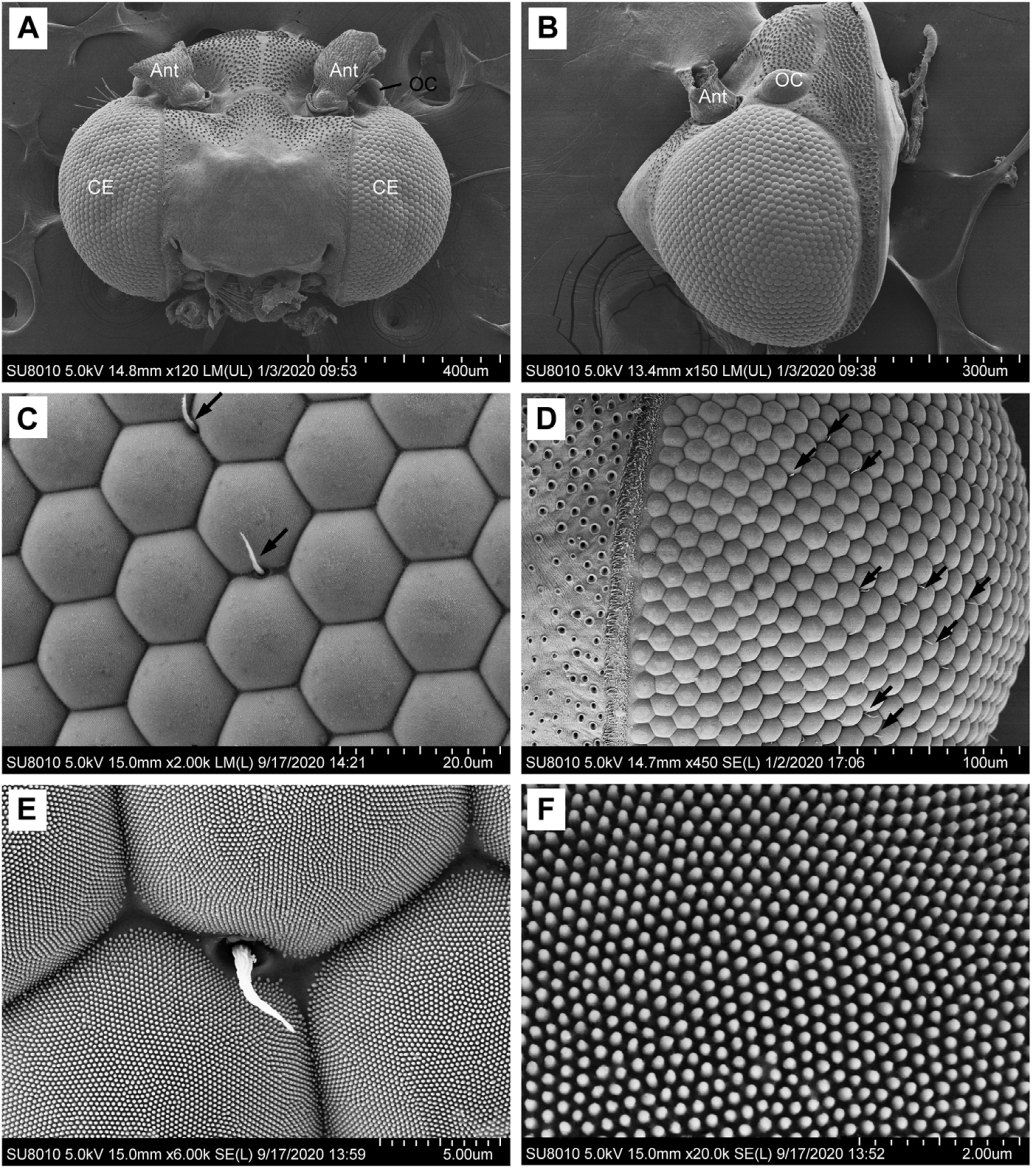
SEM micrographs of the compound eyes of male *Grapholita molesta*. **(A)** Dorsal view of the head, showing a pair of compound eyes. **(B)** Right compound eye. **(C)** Ommatidia of compound eye. Arrows indicate the interfacetal hairs. **(D) P**eripheral regions of compound eye. Arrows indicate the interfacetal hairs. **(E)** Detail of ommatidia and an interfacetal hair in the intervening line between neighbouring facets. **(F)** Corneal nipples regularly arranged in rows. Ant, antenna; CE, compound eye; OC, ocellus.

A pair of elliptical-shaped compound eyes is located on the sides of the head of adult *G. molesta* (Figure 1A). The eyes of males are significantly larger than those of the females, measuring approximately 406.2 μm in males and 392.5 μm in females along the dorso-ventral direction (*p* = 0.035), and 353.8 μm in males and 333.1 μm in females along the antero-posterior axis (*p* = 0.045). Each compound eye of male and female individuals consists of approximately 1072 and 1029 facets, respectively (*p* = 0.077), with a mean diameter of 15.2 μm (i.e., the corner to corner distance of facet; Figure 1B). Most of the facets are hexagonal in shape and arranged regularly, but some irregular shapes occur in the peripheral regions (Figures 1C, D). The corneal surface is covered with tiny corneal nipples, measuring about 256 nm in height and 101 nm in diameter. The corneal nipples are arranged regularly in a hexagonal lattice at an inter-nipple distance of 99 nm (Figure 1F). Few short interfacetal hairs (7.2 μm long and 652.8 nm in base diameter) are present at the corners or intervening lines between facets (Figure 1E). In addition to the two compound eyes, two ocelli (57.4 μm in diameter) are present (Figure 1A).

### 3.2 Internal organization

**FIGURE 2:**
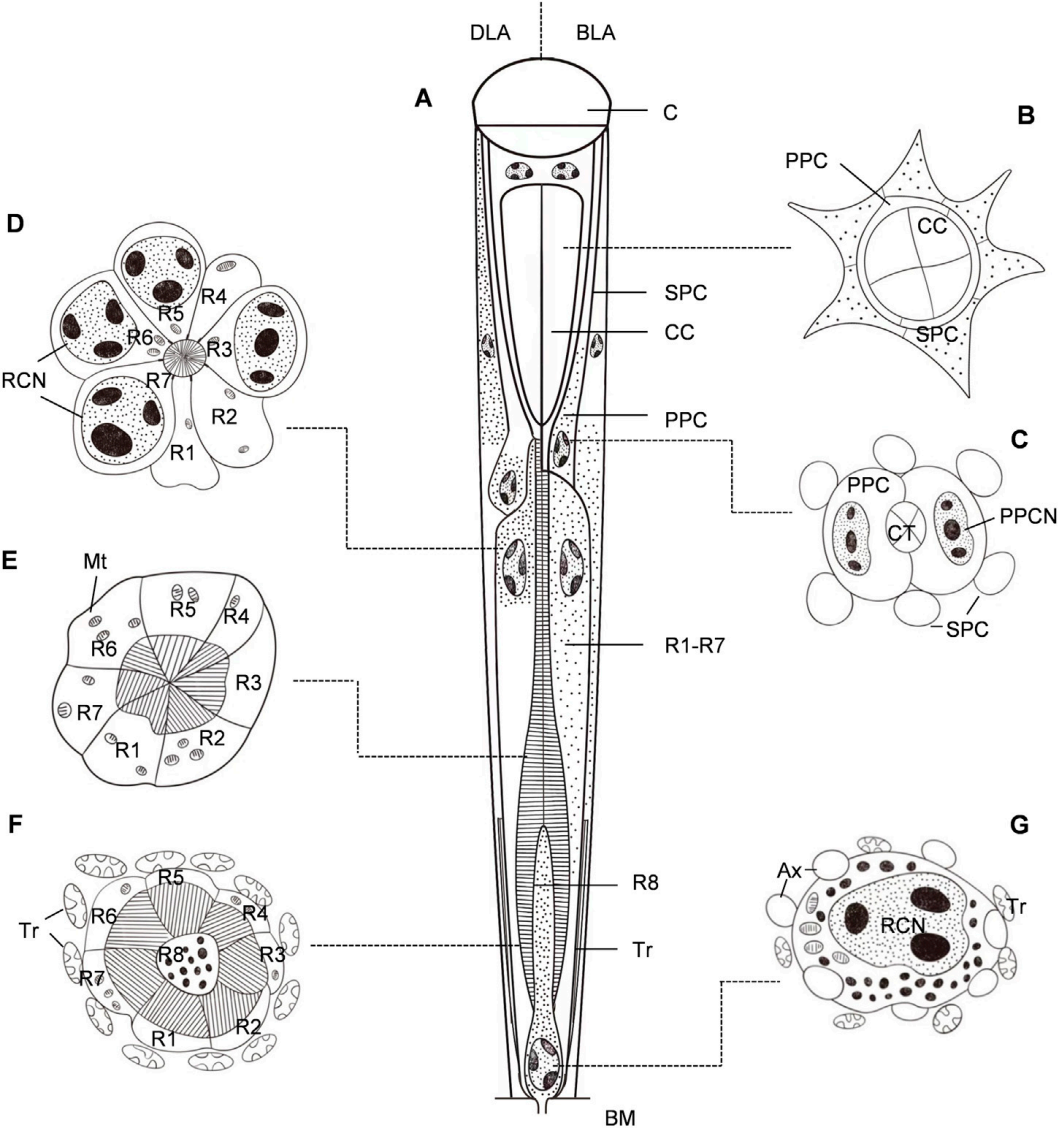
Semischematic drawings of the ommatidium of *Grapholita molesta*. **(A)** Longitudinal section of an ommatidium in dim-light-adapted (DLA; left) and bright-light-adapted (BLA; right). **(B–G)** Transverse sections of an ommatidium in bright-light-adapted at different section planes as indicated in **(A)**. **(B)** Distal region of the crystalline cone, showing the four crystalline cone cells surrounded by primary pigment cells and secondary pigment cells. **(C)** Region of the crystalline tract. The nuclei of primary pigment cells are at the proximal part of the cells. **(D)** Distal region of the rhabdom, showing seven retinula cells contribute their rhabdomeres to the rhabdom. The retinula cells contain nuclei, mitochondria and pigment granules. **(E)** Mid-region of the rhabdom, i.e., transition towards zone of proximal expansion. Few pigment granules are present in the retinula cells. **(F)** Proximal region of the rhabdom, surrounded by numerous tracheoles. The basal eighth retinula cell (R8) appeared in the centre of seven retinula cells (R1- R7) contributes its small rhabdomere to the rhabdom, distributing along the periphery of the cell in a circular shape. **(G)** Most proximal region of the retinula below the rhabdom, close to the basal matrix. The nucleus of the eighth retinula cell is located, filling nearly the entire cytoplasmic space. AX, axon; BM, basal matrix; C, cornea; CC, crystalline cone; CCN, cone cell nucleus; CT, crystalline tract; Mt, mitochondrion; PPC, primary pigment cell; PPCN, primary pigment cell nucleus; RCN, retinula cell nucleus; SPC, secondary pigment cell; Tr, tracheole.

Each ommatidium consists of the dioptric apparatus (a cornea and crystalline cone), surrounded by two primary and six secondary pigment cells and eight retinula cells. All retinula cells form a fused, bottle-shaped rhabdom, which is distally connected to the crystalline tract. Semischematic drawings of the ommatidium of *G. molesta* are illustrated in Figure 2. Based on the electron microscopic observations from the central region of the eye, no significant differences were detected between the eyes of male and female moths. Thus, the measurements were merged (Table 1). The total length of the ommatidium is on average 115.9 μm, while the average interommatidial angle is 4.3°, determined from the diameter of the corneal lens (15.8 μm) and the eye radius of 211.6 μm.

**TABLE 1:** Measurements of external and internal features of the compound eyes of *Grapholita molesta*.

Structural compositions	Morphological data	N	Unit	Measurements
Compound eyes	dorso-ventral axis length in males	5	μm	406.2 ± 2.4
	dorso-ventral axis length in females	5	μm	392.5 ± 4.8
	antero-posterior axis length in males	5	μm	353.8 ± 4.3
	antero-posterior axis length in females	5	μm	333.1 ± 6.3
	facet number in males	5	-	1072 ± 37
	facet number in females	5	-	1029 ± 15
	radius curvature eye	12	μm	211.6 ± 10.7
Corneal nipples	height	50	nm	256.0 ± 14
	diameter	50	nm	101 ± 10
	adjacent distance (in tip)	50	nm	99 ± 11
Interfacetal hairs	length	10	μm	7.2 ± 1.1
	diameter (in base)	10	nm	652.8 ± 35.4
Ommatidia	facet diameter	60	μm	15.2 ± 0.3
	length	20	μm	115.9 ± 6.0
	interommatidial angle	12	°	0.43 ± 0.4
Cornea	maximum thickness	10	μm	7.6 ± 0.4
	diameter	10	μm	15.8 ± 1.1
	number of chitin layers	8	-	30 ± 3
	radius curvature cornea	10	μm	10.8 ± 0.6
Crystalline cone	length	8	μm	35.4 ± 2.6
	diameter (distal)	10	μm	11.7 ± 1.1
Rhabdom	length	12	μm	62.3 ± 2.2
	diameter (distal)	10	μm	1.8 ± 0.3
	diameter (proximal)	10	μm	5.2 ± 0.5
	microvillus diameter	25	nm	55 ± 5
	diameter (SPC)	85	nm	378 ± 51
	diameter (retinula cells)	85	nm	349 ± 50
Basal matrix	thickness	14	nm	546 ± 64

#### 3.2.1 Dioptric apparatus

**FIGURE 3:**
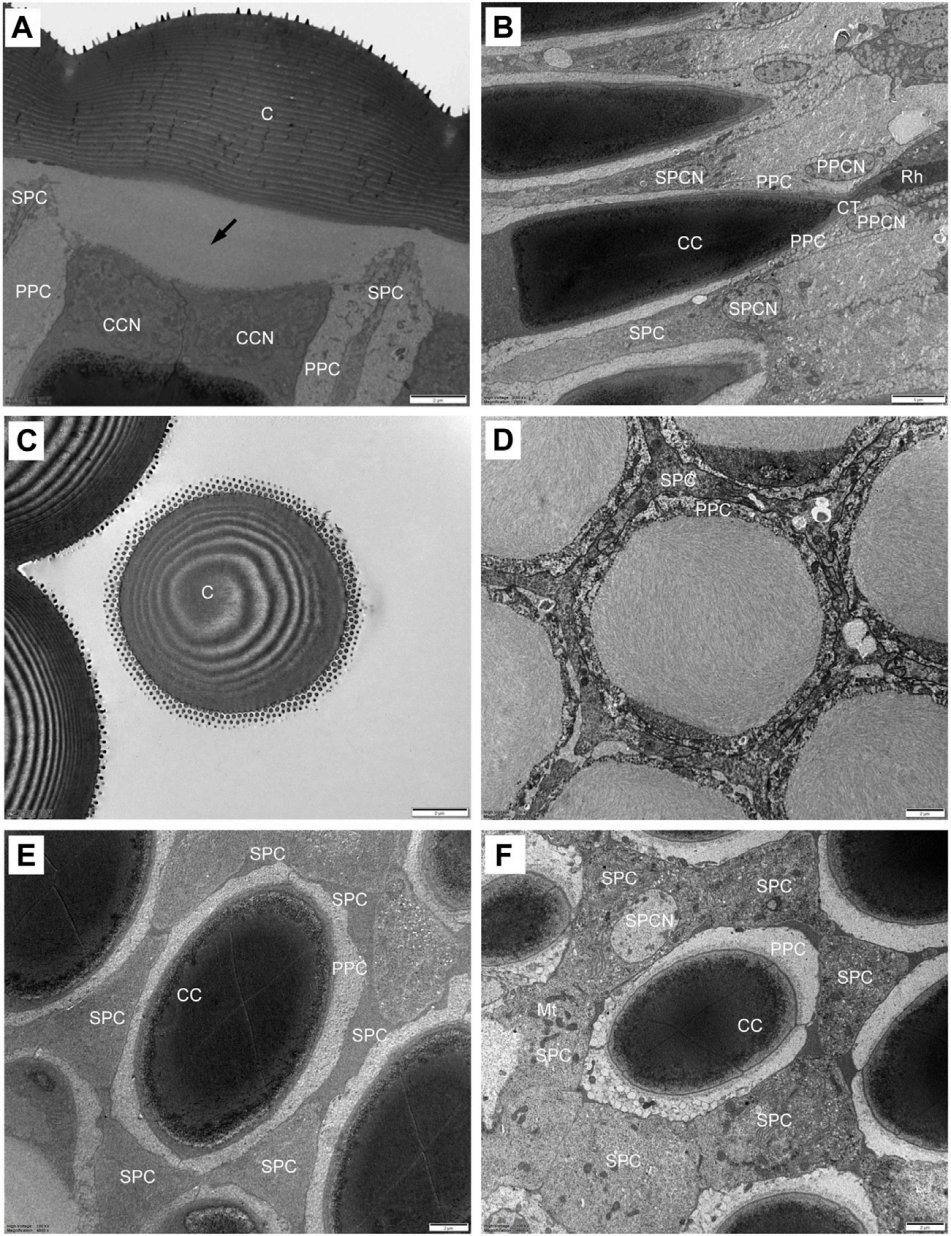
TEM micrographs showing the ultrastructure of the compound eyes of *Grapholita molesta* under bright light adaptation **(A–E)** 10000 mW/m^2^; **(F)** 100 mW/m^2^). **(A)** Longitudinal section of the laminated cornea, showing a subcorneal layer (arrows) between the cornea and the crystalline cone. **(B)** Longitudinal section of the crystalline cone and crystalline tract. **(C)** Transverse section of the laminated cornea. **(D)** Transverse section of the subcorneal layer. **(E)** Transverse section of distal region of crystalline cone, showing the four crystalline cone cells surrounded by two primary pigment cells and six secondary pigment cells. **(F)** Transverse section of proximal region of crystalline cone. The nuclei of the secondary pigment cells are visible. C, cornea; CC, crystalline cone; CCN, cone cell nucleus; CT, crystalline tract; Mt, mitochondrion; PPC, primary pigment cell; PPCN, primary pigment cell nucleus; Rh, rhabdom; SPC, secondary pigment cell; SPCN, secondary pigment cell nucleus. Scale bar: **(A–F)** = 2 μm; **(B)** = 5 μm.

The plano-convex corneal lenses possess a strongly bulged outer surface with a radius of curvature of 10.8 μm. The cornea is about 7.6 μm thick in the centre of each facet, and laminated with approximately 30 chitin layers that increase in thickness from distal to proximal regions (Figure 3A). In transverse section, the laminations become progressively tighter toward the outer side (Figure 3C). Beneath the cornea the subcorneal layer, measuring about 3.94 μm in maximum thickness can be seen. A fibrous structure that extends from the proximal region of the cornea is apparent in the subcorneal layer. The fibres are loosely arranged without any recognizable orientation (Figure 3D).

**FIGURE 4:**
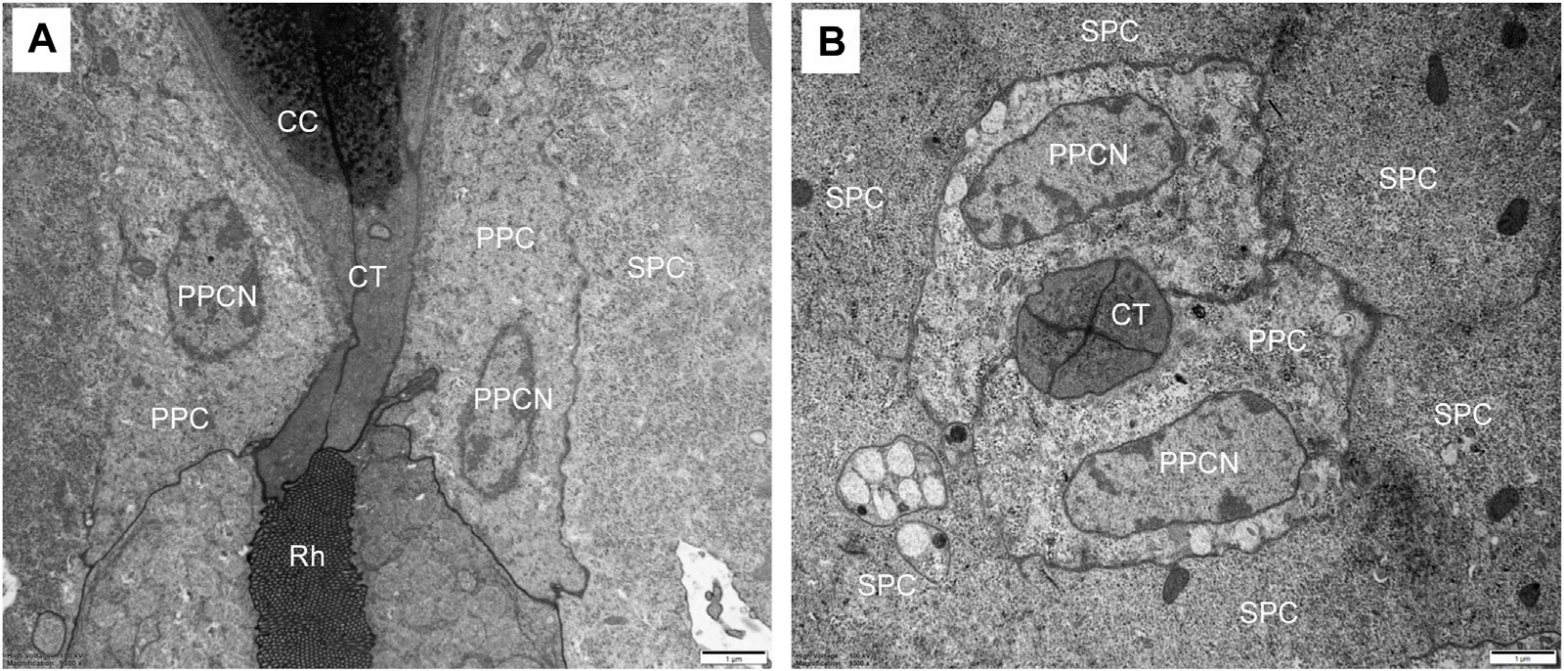
TEM micrographs of the crystalline tract of *Grapholita molesta*. **(A)** Longitudinal section of crystalline tract, which connect crystalline cone with rhabdom, showing the clear zone is absent. **(B)** Transverse section of the crystalline tract, showing the four cone cells with primary pigment cells surrounding the tract. CC, crystalline cone; CT, crystalline tract; PPC, primary pigment cell; PPCN, primary pigment cell nucleus; Rh, rhabdom; SPC, secondary pigment cell. Scale bar: **(A)** and **(B)** = 1 μm.

The crystalline cone, measuring about 35.4 μm long, is of the eucone type and formed by four cone cells. Each cone cell contain a large nuclei located in the distalmost region, but other organelles were not observed (Figures 3A, E). In longitudinal section, from its distal diameter of 11.7 μm, the bullet-shape crystalline cone gradually tapers towards the proximal end, and then forms a narrow crystalline tract (Figure 3B). The crystalline tract, less than 1.5 μm wide and 4.1 μm long, directly connects with the distal end of the rhabdom (Figures 4A, B). Microtubules are present in the crystalline tract.

#### 3.2.2 Pigment cells

Two primary pigment cells symmetrically surround both the crystalline cone and crystalline tract, and proximally contact the distal end of retinula cells. A large nucleus is located at the most proximal regions of each primary pigment cell, occupying most of the space between the crystalline tracts (Figure 3B; 4B). Peripheral to the primary pigment cells, group of six secondary pigment cells is present (Figure 3E). These cells envelop each ommatidium from the cornea down to basal matrix to fill the interommatidial spaces between neighbouring ommatidia. Being relatively small in the region of cones, secondary pigment cells broaden in the region between crystalline tract and the distal tip of the rhabdom, and then become narrow again as they proceed proximally. The secondary pigment cells contain numerous spherical electron-dense pigment granules, measuring 378 nm in diameter. The nuclei of the secondary pigment cells are located somewhat more distally, at the waist of the crystalline cone, where mitochondria are abundant in the cytoplasm of these cells (Figure 3F).

#### 3.2.3 Retinula cells and rhabdom

**FIGURE 5:**
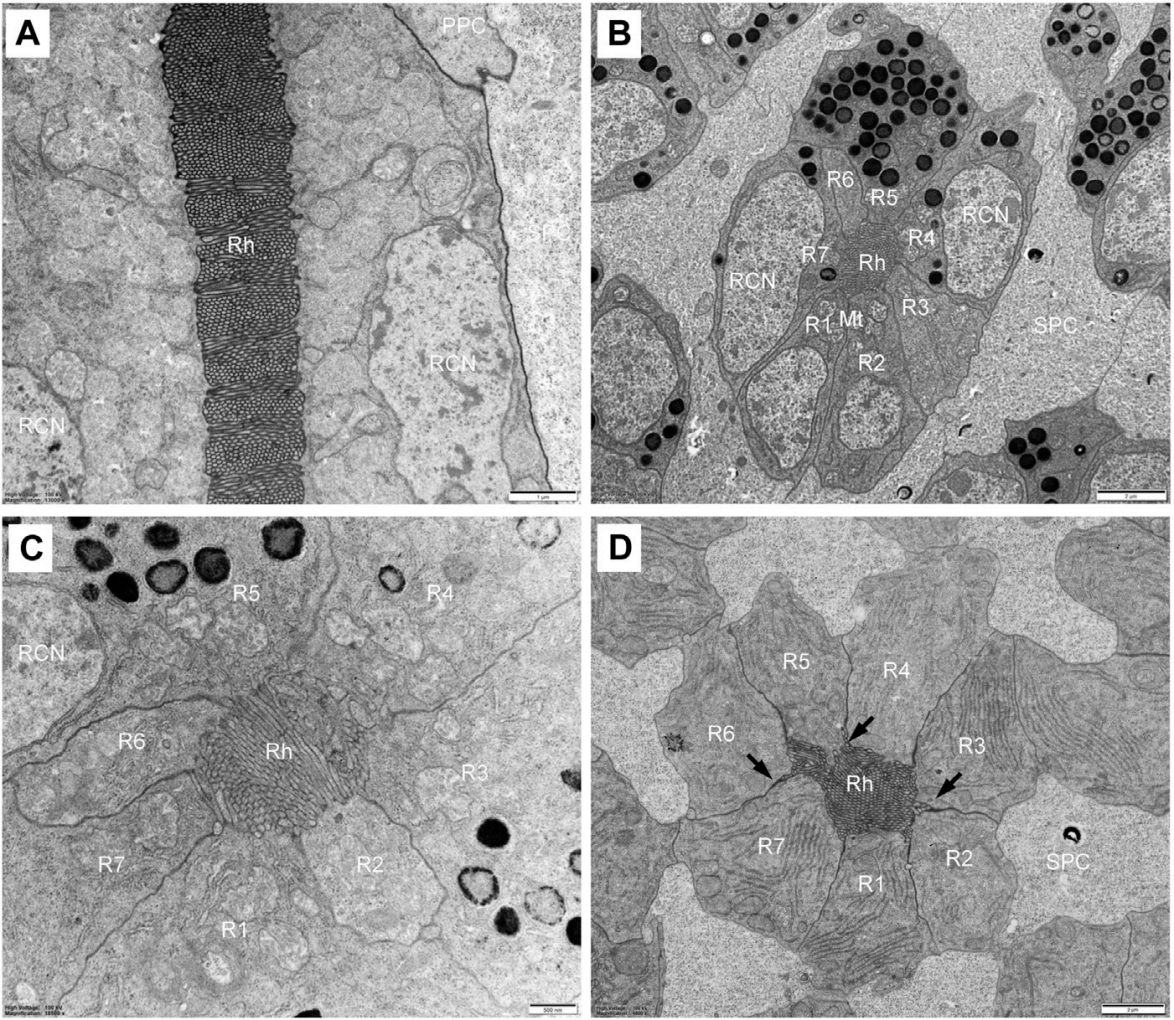
TEM micrographs of distal rhabdom of *Grapholita molesta* under bright light adaptation **(A,C** and **D)** 10000 mW/m^2^; **(B)** 100 mW/m^2^). **(A)** Longitudinal section of the distal rhabdom, showing the ordered bands of microvilli; **(B)** Transverse section of the most distal rhabdom, showing seven regular retinula cells contribute their rhabdomeres to the centrally-fused rhabdom. Pigment granules exist in the region; **(C)** Transverse section at higher magnification of the distal rhabdom; **(D)** Just below the nuclear region of seven regular retinula cells. Those retinula cells are free of pigment granules in this region. Arrowheads indicate desmosomes. Mt, mitochondrion; PPC, primary pigment cell; R1-R7, retinular cell; RCN, retinular cell nucleus; Rh, rhabdom; SPC, secondary pigment cell. Scale bar: **(A)** = 1 μm; **(B)** and **(D)** = 2 μm; **(C)** = 500 nm.

**FIGURE 6:**
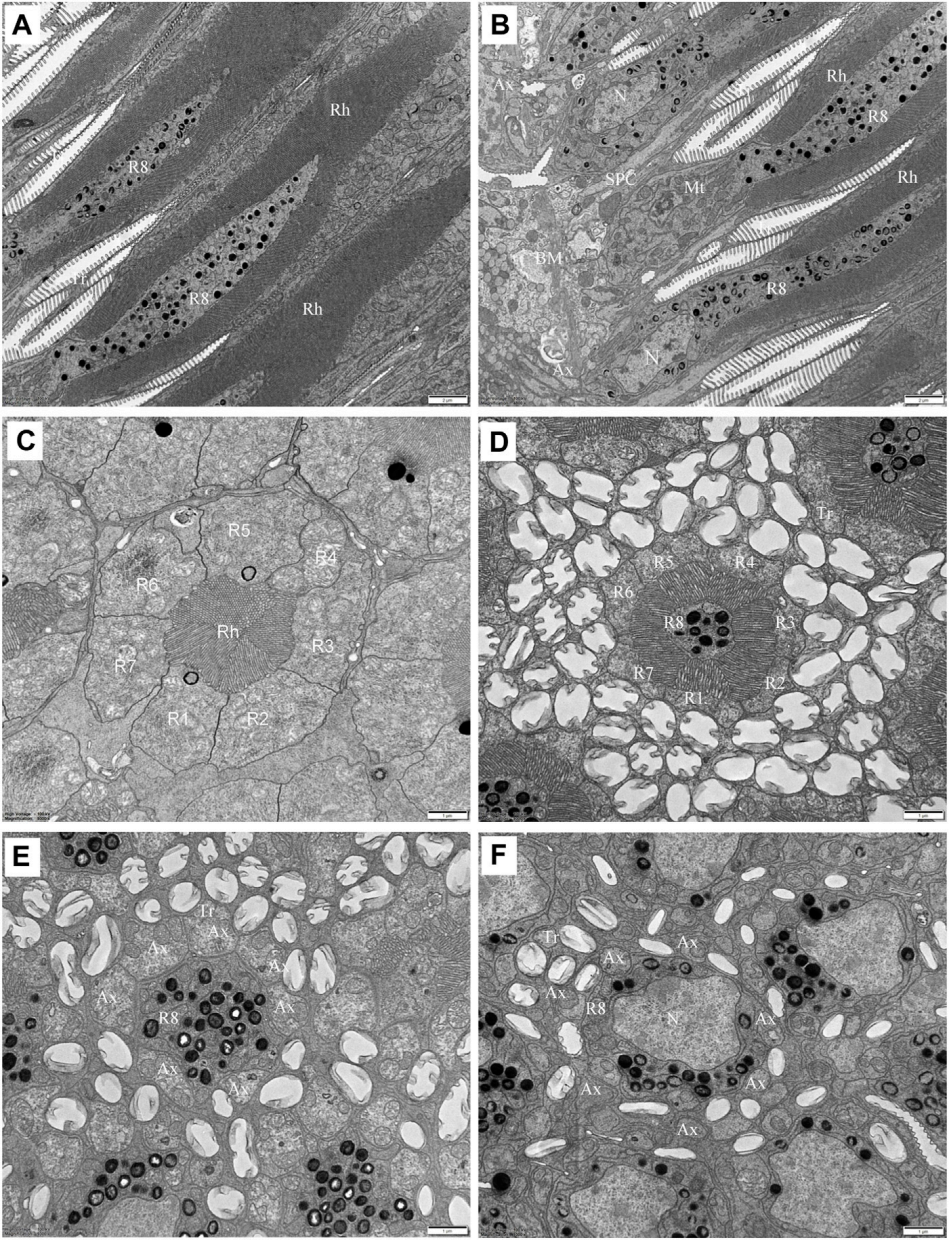
TEM micrographs of the proximal rhabdom of *Grapholita molesta* under bright light adaptation **(A,B** and **D)**10000 mW/m^2^; **(C)** 100 mW/m^2^). **(A)** Longitudinal section of the proximal rhabdom, showing the eighth retinula cell is visible centrally. **(B)** Longitudinal section of the basal region of the ommatidium. **(C)** Transverse section of the transition zone. At this region, the microvilli of those rhabdomeres become organized in different arrangements. **(D)** Transverse section of proximal rhabdom, showing pigment granules are densely distributed in the eighth retinula cell. The ommatidia are surrounded by numerous tracheoles. **(E)** The eighth retinula cell enlarges and occupies the basal region of the ommatidium immediately distal to the basal matrix. The rhabdom is completely disappeared, and other seven retinula cells turn into axons. **(F)** Nuclear region of the eighth retinula cell. AX, axon; BM, basal matrix; Mt, mitochondrion; N, nucleus; R1-R8, retinular cell; Rh, rhabdom; SPC, secondary pigment cell; Tr, tracheole. Scale bar: **(A,B)** = 2 um; **(C–E)** and **(F)** = 1 um.

**FIGURE 7:**
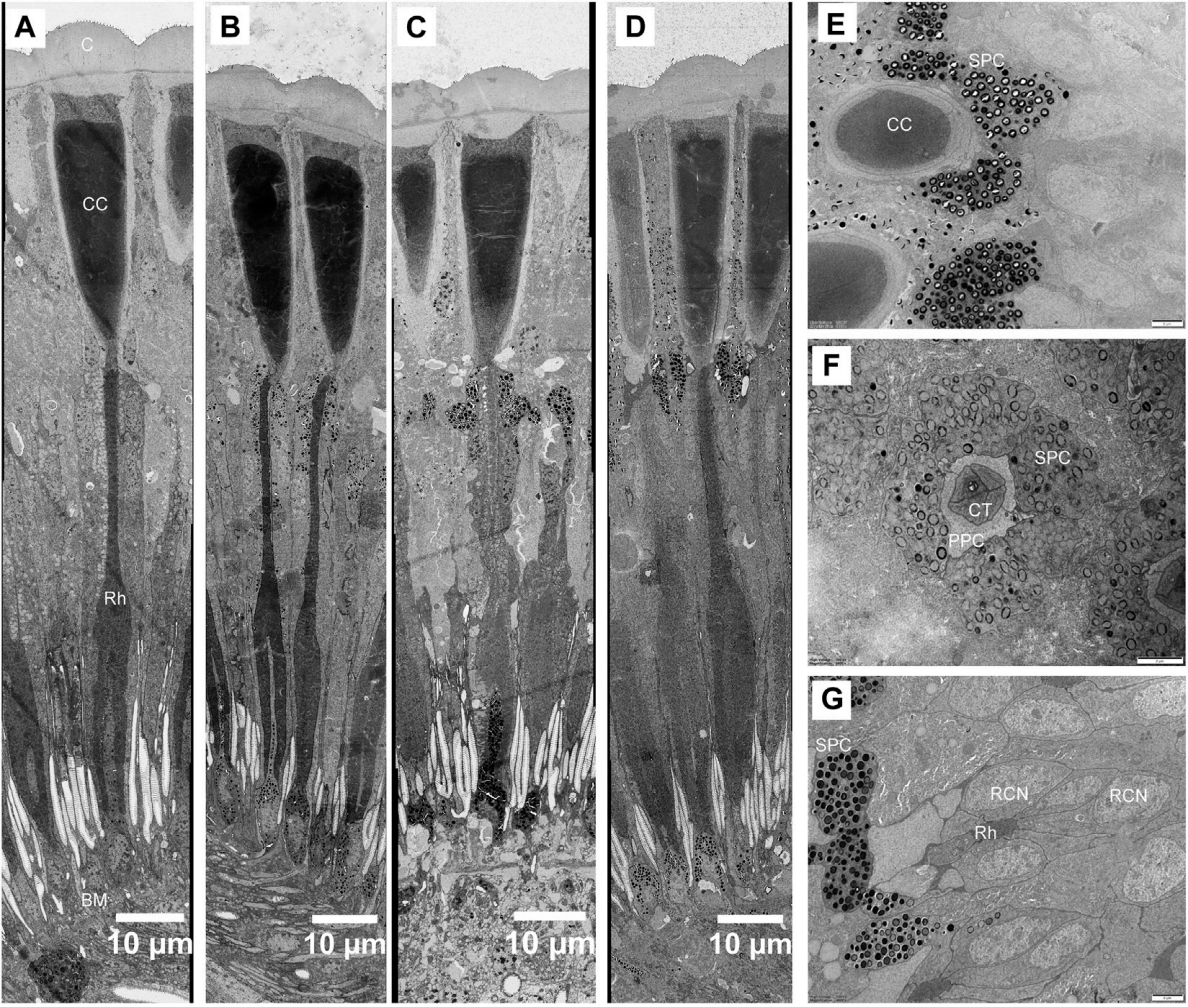
Structural changes in the compound eye of *Grapholita molesta* under different light intensity adaptation. Longitudinal section of the ommatidia exposed to **(A)** 10000 mW m^-2^; **(B)** 100 mW m^-2^; **(C)** 1 mW m^-2^; **(D)** 0.01 mW m^-2^; **(E)** Transverse section of crystalline cone under 0.01 mW m^-2^ adaptation. The pigment granules of the secondary pigment cells are restricted to surround the crystalline cone. **(F)** Transverse section of crystalline tract under 0.01 mW m^-2^ adaptation. **(G)** Transverse section of the most distal rhabdom under 0.01 mW m^-2^ adaptation. The pigment granules of the R1-R7 cells aggregated in their distal ends, close to the crystalline tract. Empty holes in section A are caused by lost pigment grains. BM, basal matrix; C, cornea; CC, crystalline cone; CT, crystalline tract; RCN, retinular cell nucleus; Rh, rhabdom; SPC, secondary pigment cell. Scale bar: **(A–D)** = 10 μm; **(E)**, **(F)** and **(G)** = 2 μm.

Each ommatidium contains eight retinula cells to form a rhabdom, measuring about 62.3 um in total length. In all ommatidia, seven retinula cells (R1-R7, called regular retinula cells) participate in the formation of the rhabdom over its entire length extending from the proximal tip of the crystalline tract down to just above the basal matrix (Figure 5A). An eighth retinula cell (R8), appearing in the centre of the rhabdom, contributes its small rhabdomere to the rhabdom only over most of the proximal part, and is referred to as the irregular retinula cell (Figure 6A). The rhabdomere of each retinula cell is made of microvilli. In longitudinal section, each rhabdom assumes a bottle shape and can be divided into two regions: a slender distal region and a wider proximal region (Figures 7A–D).

In the distal region, the R1-R7 cells are radially arranged around the longitudinal axis, and pass through the clear zone to distally connect with the dioptric apparatus (Figure 3B). Just beneath the crystalline tract, all R1-R7 cells are pigmented and form a centrally-fused distal rhabdom rod with a length of 31.4 μm and a diameter of 1.8 μm (measured in the most distal end). The microvilli of the rhabdomeres are arranged in two orthogonal directions. Parallel microvilli within a rhabdomere are oriented perpendicular to the adjacent rhabdomere, giving rise in this way to the banded appearance of the longitudinally sectioned rhabdom (Figures 5A, C). Microvilli of each rhabdomere measure about 55 nm in diameter. In this region, numerous spherical electron-dense pigment granules, about 349 nm in diameters, are present to surround the distal end of the rhabdom (Figures 5A, B). Additionally, other common organelles like mitochondria and endoplasmic reticula are distributed in the cytoplasm of the retinula cells (Figure 5D). The nuclei of R1-R7 cells, measuring about 8.9 µm in length and 2.3 µm in width, are all found at a similar level, located in the distal part of third of the retinula cells (Figures 5A, B).

In the transition zone between distal and proximal rhabdom, the rhabdom broadens and reaches its maximum diameter of 5.2 μm. It is then that the tracheal tapetum starts to surround the retinula cells. A tracheal tapetum, consisting of numerous tracheoles, extends from the basal matrix distally to the distal end of the proximal rhabdom (Figure 6A). Each ommatidium has its own tracheal tapetum so that the rhabdoms of two neighbouring ommatidia are separated by a double layer of tracheoles. At a level where the rhabdom is still fused and formed by the rhabdomeres of R1-R7, the rhabdom structure is completely different (Figure 6C). Here rhabdomeres changes into a fanshaped transverse profile with microvilli arranged in one direction, remaining the same at more proximal levels.

Further proximally, the pigment-containing R8 cell also starts to participate in the formation of the rhabdom (Figure 6B). Here, the rhabdomere of R8 is distributed along the periphery of the cell in a circular shape. In this region, the tracheoles, apposed to R8, show generally broader hollow cavities with regular internal cuticularized ridges, which optically isolate the rhabdoms (Figure 6D). Numerous mitochondria, endoplasmic reticula and multi-vesicular bodies can be seen in R1-R8 cells. All rhabdomeres gradually decrease proximally along the rhabdom and disappear about 11 μm above the basal matrix. At the proximally end of the rhabdom, the R8 enlarges and gathers pigment granules, while R1-R7 cells turn into axons (Figure 6E). The nucleus of R8 cell is located just above the basal matrix, filling nearly the entire space of the cytoplasm (Figure 6F). Finally, the retinula cell axons of each ommatidium, including that of R8 cell, penetrate the basal matrix in the form of distinct bundles of eight fibres. Some mitochondria and neurofilaments are noticeable in the axoplasm.

#### 3.2.4 Basal matrix

The basal matrix, about 546 nm in thickness, lies between the retina and the lamina, separating the optical region from neuropil (Figure 6B). Circular perforations in the basal matrix allow axon bundles of ommatidial units embedded by glial cells to pass through. Some openings in the interommatidial spaces could also be found, through which the tracheoles arising from tracheal cells below the basal matrix extend distally. In addition, large nuclei and electron-dense pigment granules are present below the basal matrix.

### 3.3 Structural changes under different light intensity adaptation

The structure of ommatidia of *G. molesta* changes under different light intensity adaptations show prominent movement of screening pigment granules (Figure 7). In dim-light-adapted eyes (1 and 0.01 mW/m^2^), the pigment granules in the secondary pigment cells migrate distally, surrounding the crystalline cone and creating a thick, electron-dense layer that extends from the cornea to the proximal tip of the crystalline cone (Figures 7C–E). The pigment granules in R1-R7 cells also move distally, concentrating in their distal ends, close to the crystalline tract (Figures 7F, G). The pigment granules move proximally upon bright-light adaptation (10000 and 100 mW/m^2^). The crystalline cone region becomes free of pigment, while the retinula cells become pigmented around the distal rhabdom (Figures 7A, B). Compared to the pigment movements occurred in secondary pigment cells and retinula cells, the pigment granules as well as nucleus in the primary pigment cells move proximally, which pigments gather around the distal end of retinula cells in dim-light-adapted eyes but scatter evenly into the cytoplasm in bright-light-adapted state. No obvious movements of pigment granules were found within the R8 cells.

The shape of crystalline tract and the position of the distal rhabdom are significant different among different light intensity adaptation (Figures 7A–D). In bright-light-adapted eyes the retinula cell bodies pull away from the cone, increasing the distance from rhabdom towards the cone and leaving behind a long narrow crystalline tract. In the dim-light-adapted eyes the length of crystalline tract decrease by more than two times. Furthermore, the proximal tips of crystalline cones are pointed in the bright-light-adapted eyes but roundish and blunt during dim-light adaptation (Figures 7A–D).

## 4 Discussion

Detailed morphological studies on the eyes of moths have been examined extensively, particularly to some moth families such as Noctuidae, Sphingidae, Pyralidae, Phycitidae, Geometridae, Crambidae, Gracillariidae and Nepticulidae (Yagi and Koyama, 1963; Horridge and Giddings, 1971; Meinecke, 1981; Lau et al., 2007; Meyer-Rochow and Lau, 2008; Honkanen and Meyer-Rochow, 2009; Fischer et al., 2012; 2014; Belušič et al., 2017; Pirih et al., 2018; Chen et al., 2019; Pan et al., 2023), ultrastructural investigations on Tortricidae, however, are still limited to just two studies, namely, *A*. *Reticulana* (Hb.) (Hämmerle and Kolb, 1987) and *Adoxophyes orana* (Fischer von Rslerstamm) (Satoh et al., 2017). The present study, therefore, provides an additional set of reference data for future studies on the lepidopteran eye.

### 4.1 Fine structural features of the eyes of G. molesta

Anatomically the compound eyes of adult *G. molest*a are of the refracting superposition eyes, indicating a high visual sensitivity to function under dim or changing light intensities at dusk. This result is confirmed by the recent works of Martín-Gabarrella et al. (2023) showing that the typical superposition deep pseudopupil can be visible in the eyes of *G. molest*a. The radius of the eye of *G. molesta* is 211.6 μm, i.e., below the theoretical limit of 250 μm to function effectively as a superposition eye (Meyer-Rochow and Gál, 2004), thus we hypothesized that the minimal limit of superposition in the eyes likely be smaller than 250 μm in radius. We will now discuss the features in the compound eyes of *G. molest*a that lead us to this conclusion.

#### 4.1.1 External morphology

Some external morphological features of compound eyes, such as the number and size of facet, are possibly essential to function effectively in dim light environments. The eyes of *G. molesta* moths consist of 1072 facets in males and 1029 facets in females with a diameter of 15.2 μm. Nocturnal *Orgyia nubilalis* (Hübner) possess a very similar facet diameter of 15 μm but a more facets (3000, Belušič et al., 2017). With larger facets in the nocturnal *Orgyia antiqua* (L.) (18.38 μm in males and 17.90 μm in females) and *Acentria ephemerella* (Denis and Schiffermüller) (15.88 in males and 17.32 μm in females), their numbers of facets, however, is less than 1000 with the exception of 2224 in male *Orgyia antiqua* (Lau et al., 2007; Lau and Meyer-Rochow, 2007).

The corneal outer surface of compound eyes in *G. molesta* features distinctly noticeable corneal nipples. With a height of 256 nm, the corneal nipples of *G. molesta* conform to the class III (nipple height around 250 nm) according to the classification described by Bernhard et al. (1970). Class III nipples in *G. molesta* is similar to those described in crepuscular and nocturnal moths, such as *A*. *ephemerella* (270 nm, Lau et al., 2007), *Ostrinia furnacalis* (Guenee) (280 nm, Chen et al., 2019) and *Heortia vitessoides* (Moore) (226.36 nm in males and 295.48 nm in females, Pan et al., 2023). Yet, the nipples in the diurnal moth *Cameraria ohridella* (Deschka and Dimic) (Fischer et al., 2012) and *Ectoedemia argyropeza* (Zeller) (Honkanen and Meyer-Rochow, 2009) are only 69.2 nm and 125 nm high, respectively, classifying as class II (nipple height 50 nm–200 nm). Since corneal nipples functions to strongly reduce the reflectance of the corneal surface, the higher nipples in crepuscular and nocturnal moths can increases the transmittance (the amount of light entering the dioptric apparatus) to enhance sensitivity in the optical system of superposition eyes and thus effectively adapt to dim-light environment (Stavenga et al., 2006).

#### 4.1.2 Dioptric apparatus

The subcorneal layer, a design generally present in diurnal butterflies (Yack et al., 2007), is found to be developed in each ommatidium of *G. molesta*. This structure is also reported in a few moths equipped with superposition eyes, such as crepuscular *Adoxophyes reticulana* (Hämmerle and Kolb, 1987) and nocturnal *O. furnacalis* (Chen et al., 2019). The maximum thickness of subcorneal layer in an ommatidium varies from species to species. It is 3.94 μm in *G. molesta*, but 4.5 μm in *O*. *furnacalis* under light and 1.5 μm under dark adaptation (Chen et al., 2019).

Produced by four cone cells, the eucone crystalline cone of *G. molesta* measures about 35.4 μm in length, which is close to that of the crepuscular moth *Acentria goedartella* (L.) (36.05 μm), and longer than of the tiny moths of the genera *Stigmella*, *Ectoedemia* and *Cameraria*, ranging from 18.19 μm to 27.0 μm (Fischer et al., 2014), but relatively short in comparison to those found in the eyes of larger species such as 45 μm in crepuscular *A. reticulana* (Hämmerle and Kolb, 1987) or 65 μm in nocturnal *O. furnacalis* (Chen et al., 2019). The crystalline cone tapers to form a crystalline tract in *G. molesta*, as found in other crepuscular and nocturnal moths including *E*. *kuehniella* (Zeller) (Horridge and Giddings, 1971), *Spodoptera exempta* (Walk.) (Meinecke, 1981), *A. reticulana* (Hämmerle and Kolb, 1987) and *O. furnacalis* (Chen et al., 2019). But their lengths are different, for example, the crystalline tract in *G. molesta* measure only 4.1 μm in length, whereas about 20 μm have been recorded in *E. kuehniella* (Horridge and Giddings, 1971) and *A. reticulana* (Hämmerle and Kolb, 1987) in the light-adapted state.

#### 4.1.3 Retinula cells and rhabdom

The ommatidia of *G. molesta* possess a distal rhabdom extension directly *c*onnected to the crystalline cone, a typical features described for apposition optics. A rather similar distal rhabdom extension has also reported in some miniature moths (body lengths of around 2–6 mm) equipped with intermediate eyes combining features of apposition and superposition optics, such as *S*. *microtheriella* (Stainton), *E*. *argyropeza*, *E*. *septembrella* (Stainton), *E*. *hannoverell*a (Glitz), *C*. *ohridella*, *Phyllonorycter esperella* (Goeze), *P*. *Maestingella* (Muller), *A*. *goedartella*, *Lyonetia clerkellaand* (L.), and *Tischeria ekebladella* (Bjerkander) (Honkanen and Meyer-Rochow, 2009; Fischer et al., 2012; Fischer et al., 2014). These moths usually possess distal rhabdom diameters greater than 2 μm. Nilsson (1989) has annotated that the thin distal rhabdom (rhabdom diameter less than 2 μm) does not inhibit the superposition process, which have confirmed by some medium-sized moths with distal rhabdom diameters of around 1 μm, inculding *E*. *kuehniella* (Horridge and Giddings, 1971), *S*. *exempta* (Meinecke, 1981), *O*. *nubilalis* (Belušič et al., 2017) and *O*. *furnacalis* (Chen et al., 2019), having perfectly functioning superposition optics. In *G. molesta* the distal rhabdom diameter of 1.8 μm lies below 2 μm, thus the superposition process remains possible.

A banded structure can be found in the distal rhabdom of G. *molesta,* likely suggesting function to detect the e-vector as reported for *O. nubilalis* (Belušič et al., 2017). Additionally, a physiological evidence for the eyes of *G. molesta* being strongly polarization sensitive has now been reported by Martín-Gabarrella et al. (2023). The polarization sensitivity in UV region of the spectrum probably mediate behaviors in *G. molesta,* inculding orientation, navigation and host recognition.

A similar arrangement of retinula cells to that in *G. molesta* has been reported in the related tortricid moths, notable *A. reticulana* (Hämmerle and Kolb, 1987) and *A. orana* (Satoh et al., 2017). Yet, eight typical retinula cells are also known from other Lepidoptera, such as *A*. *ephemerella* (Lau et al., 2007), *E. argyropeza* (Honkanen and Meyer-Rochow, 2009), *C*. *ohridella* (Fischer et al., 2012) and *A. goedartella* (Fischer et al., 2014). However, the retinula cells are arranged differently with R8 being situated at the proximal end of the rhabdom instead of the centre of the proximal rhabdom.

### 4.2 Light intensity adaptational changes in ommatidial structures

The longitudinal pigment migration provides the main structural changes in *G. molesta* during four different light intensity adaptations (10000, 100, 1, and 0.01 mW/m^2^), constituting the light-controlling pupillary mechanism. During dim-light adaptation (1 and 0.01 mW/m^2^), the pigment granules of secondary pigment cells of *G. molesta* migrate distally to surround the crystalline cone, thereby increasing light absorption by larger superposition aperture. During bright-light adaptation (10000 and 100 mW/m^2^), in turn, the proximally pigment migration into the clear zone can transform a superposition design to an apposition design to reduce light absorption. The pigment migration contributes to effectively function over a range light intensities of more than about 3 log units, adjusting the sensitivity of their eyes accordingly (Warrant and Dacke, 2011). This appears to be a common mechanism in moths with superposition eyes (Nilsson, 1989), such as *E. kuehniella* (Horridge and Giddings, 1971), *S*. *exempta* (Meinecke, 1981) and *A*. *ephemerella* (Lau et al., 2007). Compared to secondary pigment cells and retinula cells of *G. molesta,* a proximal primary pigment migration is similar to *O. furnacalis* (Chen et al., 2019), but the pigments gather around the distal end of retinula cells not crystalline tract in dark-adapted eyes.

Such pupillary mechanism often cause strong retinomotor movements affecting not only the primary pigment cells, but also the shape of the crystalline cone and the position of the distal rhabdom (Land and Fernald, 1992). From the bright-light-adapted state to the dim-light-adapted state, the pointed proximal crystalline cones in eyes of *G. molesta* turn into roundish and blunt shapes, similarly as in *C. ohridella* (Fischer et al., 2012). A change in the length characteristics of crystalline tracts during different light intensity adaptations is described in the eyes of *G. molesta*, as found in other crepuscular and nocturnal moths including *O. furnacalis* (Chen et al., 2019) and *E. kuehniella* (Horridge and Giddings, 1971)*.* In bright-light-adapted eyes a long narrow crystalline tract appears and the rhabdom distance towards the cone increases, whereas shortens the focal length and enhances the eye’s sensitivity by decreasing the length of crystalline tract in dim-light-adapted eyes of *G. molesta*. Other feathers such as the size of rhabdom are not obvious different during different light intensity adaptations.

## Data Availability

The original contributions presented in the study are included in the article/Supplementary material, further inquiries can be directed to the corresponding author.
